# Polypropylene Composites Reinforced by Nonmetallic from Waste Printed Circuit Boards Using Spout-Fluid Bed Coating with PP Particles Enhance Fluidization

**DOI:** 10.3390/polym13183106

**Published:** 2021-09-15

**Authors:** Man Wu, Jingxia Jiang, Cuiping Meng, Xiude Hu, Henglai Xie, Mingzhou Wu, Qingjie Guo

**Affiliations:** 1 Key Laboratory of Multi-Phase Fluid Reaction Engineering and Separation Engineering of Shandong Province, College of Chemical Engineering, Qingdao University of Science & Technology, Qingdao 266042, China; wuman@qust.edu.cn (M.W.); jiangjingxia10@sina.com (J.J.); m2259931221@sina.com (C.M.); henglaix@sina.com (H.X.); 2 State Key Laboratory of High-efficiency Utilization of Coal and Green Chemical Engineering, Ningxia University, Yinchuan 750021, China; xiudeh@sina.com; 3 College of Health Science and Environmental Engineering, Shenzhen Technology University, Shenzhen 518118, China; wumingzhou@sztu.edu.cn

**Keywords:** spout-fluid bed, nonmetallic materials recycled from waste printed circuit boards, coating modification, composites

## Abstract

Nonmetallic materials recycled from waste printed circuit boards (N-WPCBs) were modified by coating KH-550 in a spout-fluid bed. To improve the effect of the modification, PP particles were used to enhance the fluidization quality of the N-WPCB particles in the coating modification. Then, the modified N-WPCBs were used as fillers to fabricate PP/N-WPCB composites. The method of coating in a spout-fluid bed with PP particles enhanced fluidization and showed the best modification effect compared to other coating methods. The FT-IR and SEM results demonstrated that interfacial bonding between N-WPCBs and PP could be enhanced by modified N-WPCBs, which improved the mechanical properties of the composites. When the mass ratio of PP to N-WPCBs is 100:75 and the dose of KH-550 is 4 phr, the flexural strength, tensile strength, and impact strength of the composites increase by 16.60%, 23.22%, and 23.64%, respectively. This would realize the high-value utilization of N-WPCBs with coating modification in the spout-fluid bed.

## 1. Introduction

Printed circuit boards are a typical and fundamental component of electronic products. Globally, waste electrical and electronic equipment are one of the fastest-growing waste sectors [1]. The e-waste amount reached 53.6 million tons (Mt) worldwide in 2019, and the amount is projected to increase to 74.4 Mt by 2030 [2]. As the main component of e-waste, waste printed circuit board (WPCB) treatment has been a difficult and hot topic in the handling of e-waste. Previous research efforts have been put into the methods of separation and utilization of metal from WPCBs. However, the recycling of the nonmetallic fraction has become increasingly important due to its large volume and hazardous characteristics [3,4].

The nonmetallic materials recycled from waste printed circuit boards (N-WPCBs) were mainly composed of thermosetting resins and glass fibers [5]. The reuse of N-WPCBs to replace certain reinforcing fillers for some resin products has been studied in recent years. Commonly, N-WPCBs can be used as fillers in thermoset resin products, such as paints, wood plastic composites, adhesives, and building materials [6,7,8]. In addition to these applications, reusing N-WPCBs in general thermoplastics has great significance. Yang et al. reported the enhancement effect of N-WPCBs on the mechanical properties and heat resistance of high-density polyethylene (HDPE) [9]. Luo et al. reported that N-WPCBs were used as reinforcing fillers in rubbers. The tensile strength of rubber composites sharply increases to 18 MPa with the use of grafted polymers, which also significantly improves the thermal oxidation resistance [10]. Grigorescu et al. used N-WPCBs as fillers to improve the fire resistance of thermoplastic composites based on recycled polypropylene and diene block copolymers, and the impact strength was observed [11].

Although N-WPCBs can be successfully reused as reinforcing fillers in PP composites, the poor dispersion and weak compatibility between N-WPCBs and PP cannot be neglected [12]. Therefore, most of the N-WPCBs were modified with a coupling agent to improve the dispersion and compatibility between PP and N-WPCBs. Almost all of the modifications were carried out in a high-speed mixer [13,14]. However, due to the random contact between particles and modifier droplets in the high-speed mixer, there are some defects, such as serious particle back mixing and uneven surface coating, which restrict the modification effect of N-WPCB particles and hinder the effective utilization of modifiers. Fluidized granular coating is used to apply a shell layer onto individual core particles during fluidization. It integrates spraying, mixing, coating, and drying processes and has the advantages of high heat and mass transfer efficiency, uniform particle coverage, and continuous operation [15]. Spout-fluid beds are commonly used in coating and granulation processes because of their ability to achieve good heat transfer between solids and gases, operate in confined environments, and achieve thorough mixing [16,17].

In this paper, to improve the effect of modification, PP particles were used to reinforce the fluidization quality of the N-WPCB particles in the coating modification process. Then, the modified N-WPCBs were used as fillers to fabricate PP/N-WPCB composites. The mechanical properties of PP/N-WPCB composites and the influences of coating modification was studied. The objective of the research was to modify the N-WPCB particles in a spout-fluid bed with a draft tube, with the aim of recycling the N-WPCBs in a more profitable and environmentally friendly way.

## 2. Materials and Methods

### 2.1. Materials

**Figure 1:**
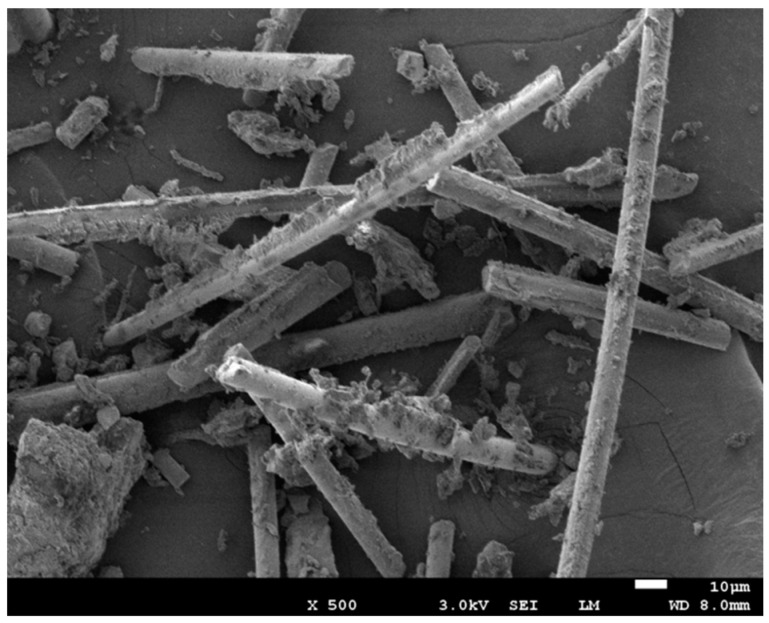
SEM images of N-WPCBs.

The WPCBs used in this study were provided by a company specializing in hazardous waste disposal in Henan Province, China. WPCBs were ground to less than 0.2 mm to remove the metal. The average particle size of N-WPCBs was 95 μm, which was measured by laser diffraction particle size analyzer (Rise-2002, Jinan Runzhi Technology Co., Ltd., Jinan City, Shandong Province, China). The particle density was 2165 kg·m^−3^. The SEM of N-WPCBs is shown in Figure 1.

The PP particles were collected from Liaoni Huajin Tongda Chemicals Company, China. In the fluidization coating process, these PP particles were used as auxiliary fluidizing particles with a particle size of 450 μm and a particle density of 843 kg·m^−3^. Meanwhile, in the PP/N-WPCB composite, PP was the matrix material with a MFR of 4.25 g·(10 min)^−1^. The γ-aminopropyl triethoxysilane (KH-550) was supported by Qingdao Xuxin Chemical Co., Ltd., Qingdao City, Shandong Province, China. The density of KH-550 was 946 kg·m^−3^ with a molar mass of 221.4 g·mol^−1^. The pentaerythritol tetrakys 3-(3,5-ditert-butyl-4-hydroxyphenyl) propionate (antioxidant 1010), tns-(2.4-di-tert-butyl)-phosphite (antioxidant 168), stearic acid, and zinc stearate were provided by Qingdao Zhengfang Plastic Co., Ltd., Qingdao City, Shandong Province, China.

### 2.2. Spout-Fluid Bed Coating Device and Procedure

**Figure 2:**
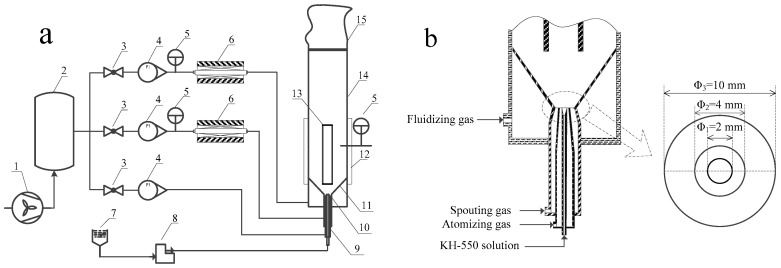
(**a**) Flow chart of the experiment apparatus, (**b**) Atomizer and spout gas inlet. 1. Root blower, 2. Buffer tank, 3. Pilot valve, 4. Rotameter, 5. Temperature controller, 6. Preheater, 7. Storage tank, 8. Peristaltic pump, 9. Annular jet nozzle, 10. Spouted inlet, 11. Distribution plate, 12. Heater band, 13. Draft tube, 14. Spout-fluid bed, 15. Bag-type dust remover.

The N-WPCB particle was coated in a spout-fluid bed with a draft tube. Figure 2a shows that the experimental consists of a spout-fluid bed, gas supply system, liquid atomizing system, temperature adjusting system, pressure signal sampling system, and processing system. The main component of the spout-fluid bed is a stainless-steel column with inner diameter of 160 mm and height of 1400 mm. A conical gas distributor with a 60° inclination angle is located at the bottom of the column. The orifices with inner diameter of 1 mm are vertically distributed on the gas distributor with hole ratio of 1%. Two stainless steel layers of 48 μm mesh are covered on the gas distributor in order to evenly distribute the gas and prevent leakage particles. A draft tube with a height of 450 mm and a diameter of 45 mm is mounted axially above the spout inlet nozzle. The distance between the spout inlet nozzle and the bottom of draft tube is 50 mm. The atomizer and spout gas inlet are installed at the bottom of conical gas distributor, and the structure and size are shown in Figure 2b.

During the fluidized coating process, the N-WPCB particles were mixed with PP, which was used as the auxiliary fluidizing particle. Then, the PP and N-WPCB mixed particles were placed in the bed and fully fluidized by the preheated spouting gas and fluidizing gas. Once the temperature in the bed reached the preset temperature, the coupling agent solution was injected into the bed by the nozzle. The N-WPCB particles were coated by contacting and impacting droplets near the atomizer. The wet particles were dried by continuing fluidization at the preset temperature. Then, N-WPCB particles were withdrawn from the spout-fluid bed.

During the process of particle coating, the pressure drops throughout the bed were measured using a differential pressure sampling apparatus in order to evaluate the fluidization quality of PP and N-WPCB binary mixed particles. Pressure-measuring holes were connected to differential pressure transducers (Oute LD 185B, Yantai, China). The total pressure drop of the bed was Δ*P*_T_, and the pressure drop of 100 mm in the annular region was marked Δ*P*_A_. Δ*P*_T_ and Δ*P*_A_ represent the average values of the sampling data. The behavior of the particles was observed through the visible windows of the column.

### 2.3. Preparation of the PP/N-WPCBs Composites

In the process of PP/N-WPCBs composite preparation, the mixture of PP matrix, modified N-WPCB particles (40 phr), antioxidant 1010 (0.5 phr), antioxidant 168 (0.5 phr), stearic acid (0.5 phr), and zinc stearate (0.5 phr) was extruded in a double-screw extruder (SHJ-20, Nanjing GIANT Machinery Co., Ltd., Nanjing City, China), and then cut into composite particles using a pelletizer. The composite particles were dried for 4 h at 80 °C. Using the dried composite particles, the standard specimens were made by injection molding with an injection machine (130F2V, Huadong Machinery Co., Ltd., Qingdao City, Shandong Province, China). The mechanical properties of the specimen were tested after 16 h.

### 2.4. Measurement of Properties

The tensile and flexural properties of the PP/N-WPCB composites were determined according to ISO 527 and ISO 178 standards using a computer servo universal testing machine (GT-10S-2000, GOTECH testing Machines Inc., Taiwan) at cross-head speeds of 2 mm·min^−1^ and 10 mm·min^−1^, respectively. Five specimens were tested for each type of composite, and then the results were averaged. A notched impact test was carried out according to ISO 180 standards using an Izod impact testing machine (XCT-40, Chengde Precision Tester Co., Ltd., Chengde City, Hebei Province, China) with a 1 J pendulum. Ten specimens were tested for each type of composite, then averaged the results.

The melting and crystallization behavior of composites samples were studied through differential scanning calorimetry (DSC Q20, TA Instruments, USA). For this process, 5–10 mg of the sample was weighed very accurately. The samples were held at 200 °C for 5 min to remove the thermal and mechanical history. Then, the crystallization and melting curves were obtained by cooling the samples from 200 to 30 °C at a rate of 10 °C·min^−1^ and heating the samples back to 200 °C at 10 °C·min^−1^, respectively. The crystalline ratio $X_{c}$ was calculated by the following Equation (1) [18]:

(1) $${\;X}_{c}=\frac{\Delta H_{m}}{\left(1-\varphi \right)\Delta H_{c}^{0}}$$

where $\Delta H_{m}$ is the melting enthalpy of samples, $\Delta H_{c}^{0}$ is the theoretical value of the melting enthalpy for a 100% crystalline PP, which is 209 J·g^−1^, and *φ* is the weight fraction of WPCB in the sample.

Field-emission scanning electron microscopy (JSM-6700F, JEOL Co., Tokushima, Tokyo, Japan) was used to analyze the dispersion of the fillers in the resin matrix and the interfacial bonding degree between the fillers and the resin using the fracture surfaces. Prior to analysis, the fractured surfaces of the specimens were sputter coated with a thin layer of gold. Fourier transform infrared spectroscopy was conducted using a Fourier infrared spectrum analyzer (Tensor-27, Bruker Co., Karlsruhe, Germany) to verify the surface functional groups of the N-WPCB particles before and after modification with the coupling agents.

## 3. Results and Discussion

### 3.1. Effect of Auxiliary Fluidized Particles on the Mechanical Properties of Composites

#### 3.1.1. Improving Fluidization by Adding PP Particles

**Figure 3:**
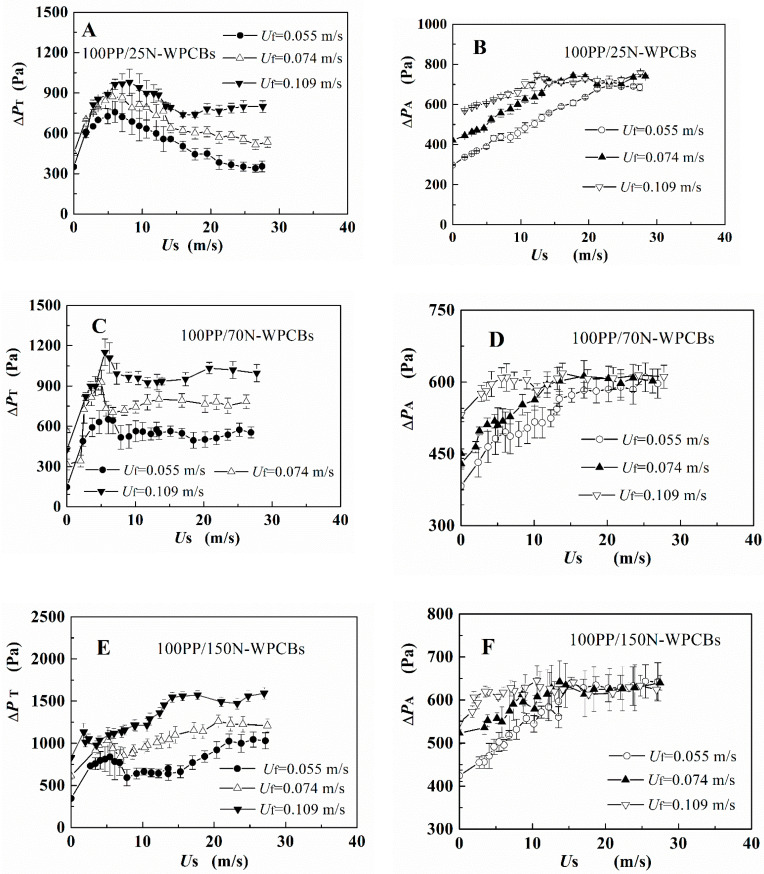

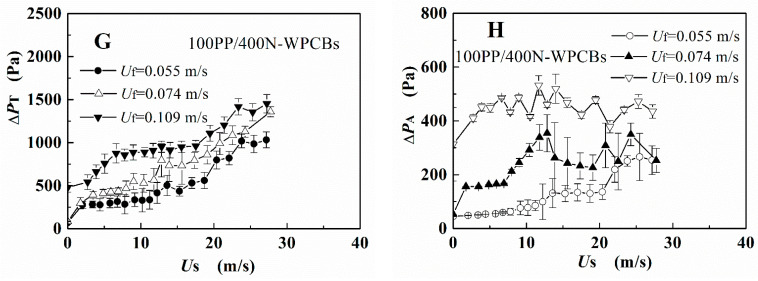
Results of the total pressure drop Δ*P*_T_ and pressure drop in the annular region Δ*P*_A_ by decreasing the spouting gas velocities with the addition of fluidizing gas. (**A**): Total pressure drop Δ*P*_T_ for 100PP/25N-WPCBs ; (**B**): Pressure drop in annular region Δ*P*_A_ for 100PP/25N-WPCBs ; (**C**): Total pressure drop Δ*P*_T_ for 100PP/70N-WPCBs ; (**D**:) Pressure drop in annular region Δ*P*_A_ for 100PP/70N-WPCBs ; (**E**): Total pressure drop Δ*P*_T_ for 100PP/150N-WPCBs ; (**F**): Pressure drop in annular region Δ*P*_A_ for 100PP/150N-WPCBs ; (**G**): Total pressure drop Δ*P*_T_ for 100PP/400N-WPCBs ; (**H**): Pressure drop in annular region Δ*P*_A_ for 100PP/400N-WPCBs.

The irregular N-WPCB particles exhibited poor flowability and could not be directly spouted or fluidized. Thus, PP particles were used as the auxiliary fluidization medium [17]. With spouting and fluidizing gas velocities, the pressure drop evolution for the four groups of binary mixed particles 100PP/25N-WPCBs, 100PP/70N-WPCBs, 100PP/150N-WPCBs, and 100PP/400N-WPCBs are shown in Figure 3. In each run, the evolution of the pressure drop with spouting gas velocity was monitored from the spouting state to the fixed bed by holding a given fluidizing gas.

For particle mixtures 100PP/25N-WPCBs, 100PP/70N-WPCBs, and 100PP/150N-WPCBs, the pressure drops Δ*P*_T_ and Δ*P*_A_ initially increased to a maximum value as the spouting gas velocity increased in the condition of adding fluidizing gas in the annular area. When the spouting gas velocity continued to increase, all the pressure drops stabilized for the 100PP/25N-WPCB and 100PP/70N-WPCB particle mixtures. However, Δ*P*_T_ displayed slight increases for the 100PP/150N-WPCB mixture. Combined with each test experiment phenomena, a large number of particles spouted from the draft tube at a high spouting gas velocity. The particle mixtures stopped spouting when the total pressure drop Δ*P*_T_ reached the maximum value with the decrease of the spouting gas. For the 100PP/400N-WPCB particle mixture, the total pressure drop ΔPT had an increasing trend over the whole range of spouting gas velocities. Meanwhile, some channeling and dead zone could be observed in the bed. These undesirable fluidization phenomena did not disappear even with the increase of gas velocity. 

With the change of fluidizing gas, the three group binary mixtures 100PP/25N-WPCBs, 100PP/70N-WPCBs, and 100PP/150N-WPCBs showed similar pressure drop trends. That is, after the fountain was formed in the bed, the total pressure drop increased with the increasing of the fluidizing gas. Meanwhile, the particle circulation also increased with the increasing of fluidizing gas velocity. This is similar to previous reports for spout-fluid beds with or without a draft tube [19]. Therefore, for these three group particle mixtures, the binary mixtures showed good fluid-dynamic stability. The good fluidization quality of the PP/N-WPCB mixtures could improve the modification of the N-WPCB particles during the coating of KH-550.

#### 3.1.2. Effect of the Fluidized Particle Ratio on the Mechanical Properties of Composites

**Figure 4:**
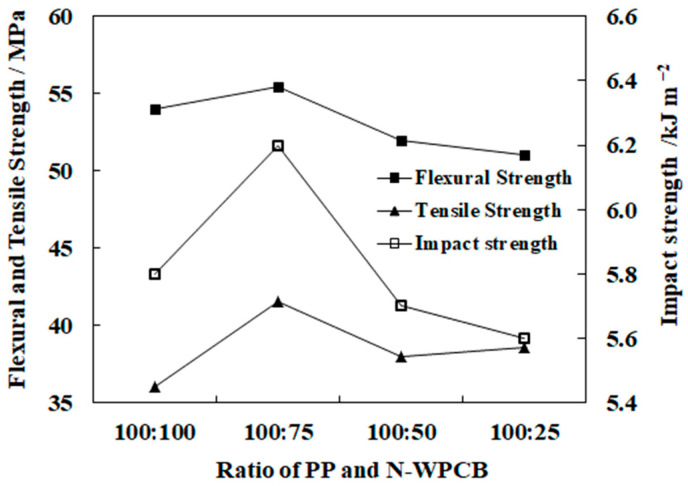
Effect of the PP content of the mixed particles on the mechanical properties of composites.

During fluidization coating, the PP particles were used to assist the fluidization medium, which improved the fluidization quality [20]. The effects of the PP content of the mixed particles on the mechanical properties are shown in Figure 4. The flexural strength, tensile strength, and impact strength of the composites first increased and then decreased with increasing mass ratio of PP to N-WPCBs, and the best mass ratio was 100:75.

During the experiments, the fluidization behavior of the cohesive N-WPCB particles was improved by the PP particles. On the one hand, assistant particles could reduce the Hausner ratio (Hr, the ratio of tapping density to bulk density, a measurement of the viscosity) of the cohesive particles. On the other hand, assistant particles could break the particle agglomerates by high-speed crashes [21,22]. The improvement in the quality of the fluidization of N-WPCB particles could improve the modification effect of the N-WPCB particles and the mechanical properties of the PP/N-WPCB composites. However, when the PP content of the mixed particles was too high, a large amount of KH-550 adhered to the surface of the PP particles and resulted in the waste of KH-550. When the PP content of the mixed particles was too low, the fluidization quality of the mixed particles was poor. Thus, an optimal mass ratio of PP particles to N-WPCB particles should be selected.

### 3.2. Effect of Spouting Gas on the Mechanical Properties of PP/N-WPCB Composites

**Figure 5:**
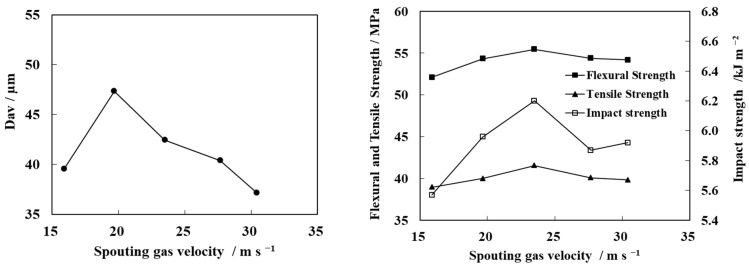
Effect of spouting gas velocity on the average particle size of modified N-WPCBs and the mechanical properties of PP/N-WPCB composites.

The spouting gas is the key factor for the fluidization of N-WPCB particles in the bed. It can further affect the modification of N-WPCB-coated particles and the mechanical properties of PP/N-WPCB composites. The change in the modified N-WPCB particle size and mechanical properties of the PP/N-WPCB composite with spouting gas velocity is shown in Figure 5. The modified N-WPCB particle size and the mechanical properties of the composites first increase and then decrease with increasing spouting gas velocity. The optimal spouting gas velocity is 23.49 m·s^−1^.

At a low spouting gas velocity, the fluidization of particles in the bed is unstable, which leads to the aggravation of particle agglomeration growth, resulting in a large particle size after coating [23]. Due to the poor N-WPCB surface modification process, the mechanical properties of the composites were low. With an increasing spouting gas velocity, the breaking ability of particle agglomerates was enhanced due to the spouting gas jet [24]. Under this condition, the growth mode of particles was mainly layer growth, which is conducive to the surface modification of particles. In addition, the heat and mass transfer efficiency is further improved, which is conducive to the uniform coating of the modifier on the surface of the particles and for improving the modification effect of the particles [25]. Hence, the modified particle size reduced. Meanwhile, the mechanical properties of the composites increased. However, when the spouting gas velocity continued to increase, some droplets were taken out of the bed by the jet before colliding with the particles, resulting in the loss of the modifier and the modification effect of N-WPCB particles, which led to a decrease in the mechanical properties of the composite material.

### 3.3. Effect of KH-550 Content on the Mechanical Properties of Composites

**Figure 6:**
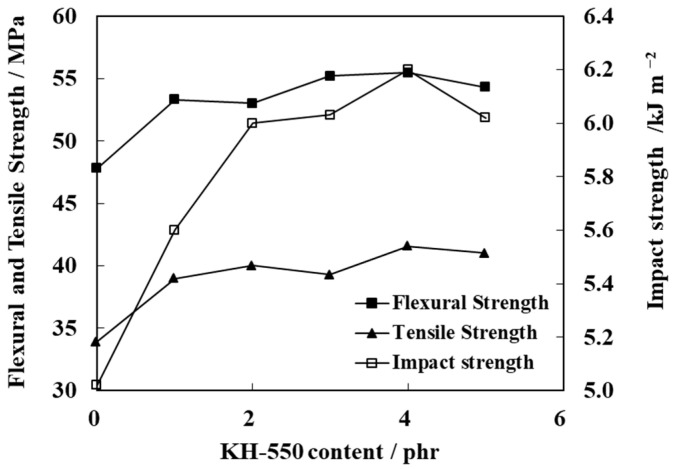
Effect of the KH-550 content on the mechanical properties of PP/N-WPCB composites.

The effect of KH-550 content on the mechanical properties of PP/N-WPCB composites is shown in Figure 6. The mechanical properties of the composites are obviously improved by coating with KH-550. With the increasing dose of KH-550, the mechanical properties of the composites first increase, and then the flexural strength and impact strength show slightly downward trends. The optimum dose of KH-550 is 4 phr (phr, the ratio of KH-550 to N-WPCBs).

With the addition of KH-550, both the hydrophobic and hydrophilic ends of KH-550 interacted with the N-WPCB particles and PP matrix at the same time. Tight bonding between the particles and the matrix occurred. Therefore, the mechanical properties of the composites were improved by KH-550. With the increasing in the addition of KH-550, the surface area of the modified N-WPCB particles increased, and a more complete interface formed between the modified N-WPCBs and PP. Stress could easily transfer from the PP matrix to N-WPCB particles, resulting in an improvement in the mechanical properties of the composites [26]. Nevertheless, when the dose of KH-550 exceeded 4 phr, excess silanol (produced by KH-550 hydrolysation) accumulated on the surface of N-WPCB particles, acting as a stripping agent or lubricant [27]. The N-WPCB particles were easily stripped from the PP matrix. Therefore, the composites easily broke under the external force.

### 3.4. Effect of Droplet Size on the Mechanical Properties of PP/N-WPCB Composites

#### 3.4.1. Effect of Spray Rate on the Mechanical Properties of PP/N-WPCB Composites

**Figure 7:**
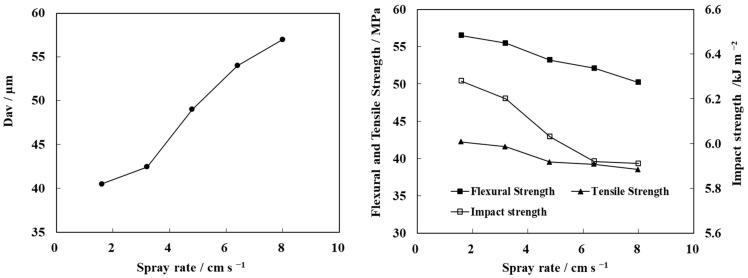
Effect of spray rate on the average particle size of modified N-WPCBs and the mechanical properties of PP/N-WPCB composites.

The spray rate has a direct effect on droplet size and uniformity [28]. Keeping the total amount of liquid constant, the effect of spray rate on the mechanical properties of PP/N-WPCB composites was studied. As shown in Figure 7, the modified particle size increased with the increasing of spray rate, while the mechanical properties of the composites decreased. This is because the droplet diameter increases with increasing spray rate when the solution is torn in the nozzle. The large droplet diameter leads to an increase in the probability of bonding between wet particles, accelerating the growth rate of modified particles. Eventually, the modified particle size is increased. However, the large spray rate more easily strengthened the growth of modified particles by agglomeration in the coating process, which reduced the uniformity of the modified particle coating [29]. Under this circumstance, the particle modification effect and the mechanical properties of the materials decreased. The low spray rate of the modifier solution can enhance the modification effect of particles in spout-fluid beds. However, the coating time was prolonged, resulting in an increase in energy consumption. When the spray rate increased from 1.6 cm·s^−1^ to 3.2 cm·s^−1^, the coating time was reduced by half. In addition, the bending, tensile, and impact strength of the material only decreased by 2.14%, 0.87%, and 1.81%, respectively. As the spray rate continued to increase to 4.8 cm·s^−1^, an agglomeration phenomenon appeared around the nozzles, while some particles adhered on the inner wall. This is because the wetting rate of the particle surface was much higher than that of the drying rate. Therefore, the best spray rate was 3.2 cm·s^−1^ in the next step.

#### 3.4.2. Effect of Atomizing Gas Velocity on the Mechanical Properties of PP/N-WPCB Composites

**Figure 8:**
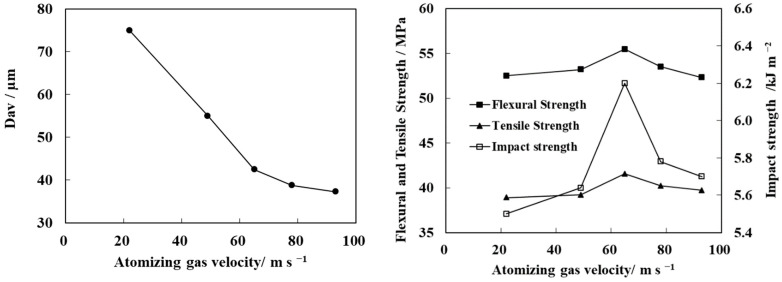
Effect of atomizing gas velocity on the average particle size of modified N-WPCB and the mechanical properties of PP/N-WPCB composites.

The atomization gas velocity is another key factor of the droplet size. The change in the modified N-WPCB particle size and mechanical properties of the PP/N-WPCB composite with atomization gas velocity is shown in Figure 8. At a low atomization gas velocity, the modified particle size decreases significantly with the increase of atomization gas velocity. When the atomization gas velocity exceeds 65.08 m·s^−1^, the rate of decrease of the modified particle size slows down. The bending strength, tensile strength, and impact strength of the composites first increase and then decrease with the increasing of atomization gas velocity. When the atomization gas velocity is 65.08 m·s^−1^, the mechanical properties of composites are the best.

With increasing atomization gas velocity, the average diameter of droplets decreased, and the distribution of droplet size was uniform [30]. The modified particle growth mode changed from agglomeration to layer growth with decreasing droplet diameter. Therefore, the size of modified particles decreased. At this time, the decrease in droplet diameter increased the chance of collision between particles and droplets, which was conducive to improving the effective capture of particles to the modifier droplets. Meanwhile, the high drying rate of the particle surface improved the coating quality of the particle uniformity. However, when the droplet diameter was too small, some droplets dried and solidified before colliding with the particles. In this case, an effective coating could not form. In conclusion, there was a suitable atomization gas velocity that optimized the particle modification effect. Meanwhile, the mechanical properties of the material were also high.

### 3.5. Effect of Temperature on the Mechanical Properties of PP/N-WPCB Composites

**Figure 9:**
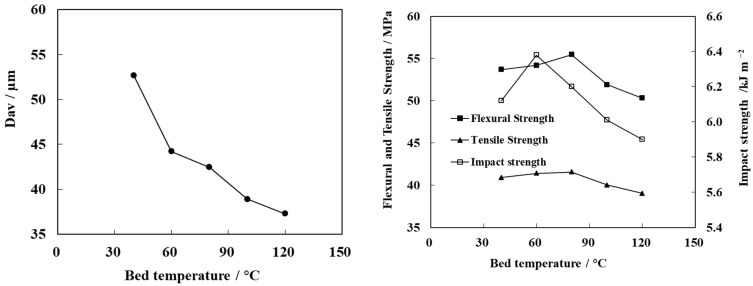
Effect of bed temperature on the average particle size of modified N-WPCBs and the mechanical properties of PP/N-WPCB composites.

The modification of particles in the spout-fluid bed is closely related to the drying rate of the particle surface. Temperature is the factor controlling the drying rate. The changes in the modified particle size and mechanical properties of the PP/N-WPCB composite with bed temperature are shown in Figure 9. With an increasing bed temperature, the modified particle size decreases. The mechanical strength of the composites first increases and then decreases, and the optimal bed temperature is 80 °C.

At a low temperature, the drying rate of particles increased with increasing temperature. Under this condition, the growth mode of particles changed from agglomeration to layer growth. The modified particle size was reduced with the inhibition of agglomeration, which was conducive to the uniform coating of modified particles on the surface [31]. Therefore, the mechanical strength of the composites also increased. However, when the temperature was over 80 °C, the evaporation rate of the solution was too fast. This resulted in the solidification of modifier droplets before they reached the surface of particles [32]. The utilization rate of the modifier decreased. In conclusion, high or low temperatures reduce the modification effect of particles, resulting in a decrease in the mechanical properties of composites. When the bed temperature was 60~80 °C, the wetting rate and drying rate of the particle surface reached a balance, which maximized the particle modification effect. The mechanical properties of the composite also reached the optimal values.

### 3.6. Characterization of KH-550 Modification

To study the modification mechanism of KH-550, the variations in the surface functional groups and the cross-section morphology were characterized by infrared spectroscopy and scanning electron microscopy. 

#### 3.6.1. SEM Images of PP/N-WPCB Composites

**Figure 10:**
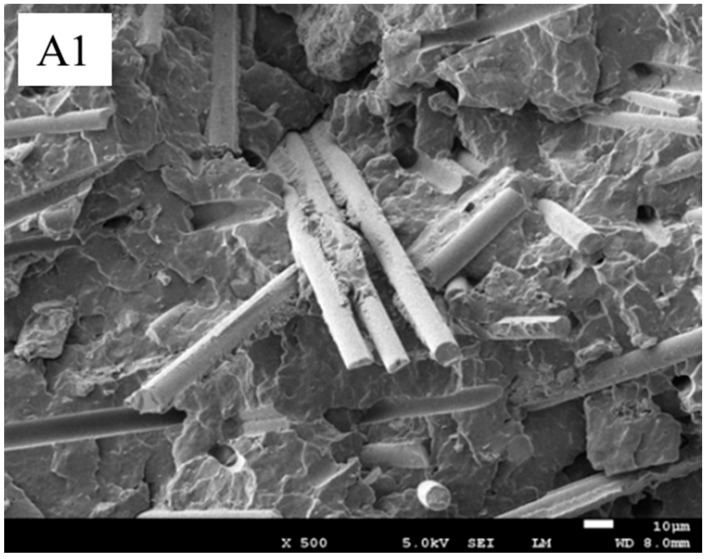

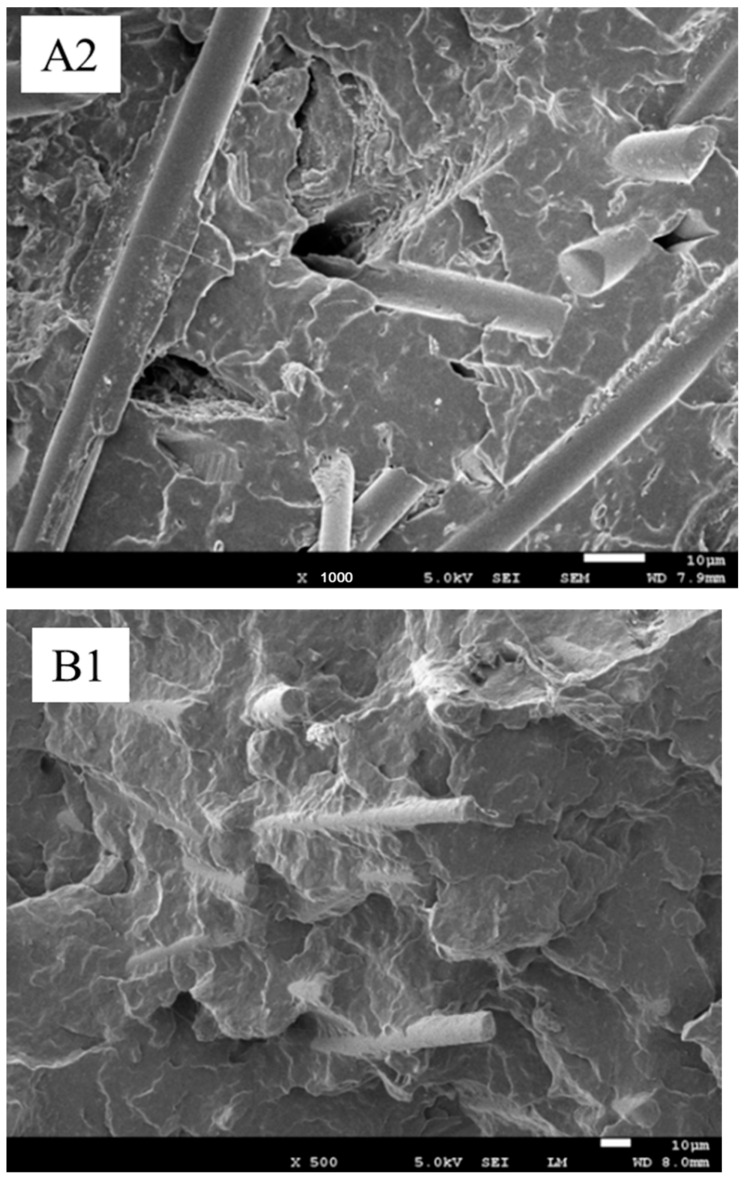

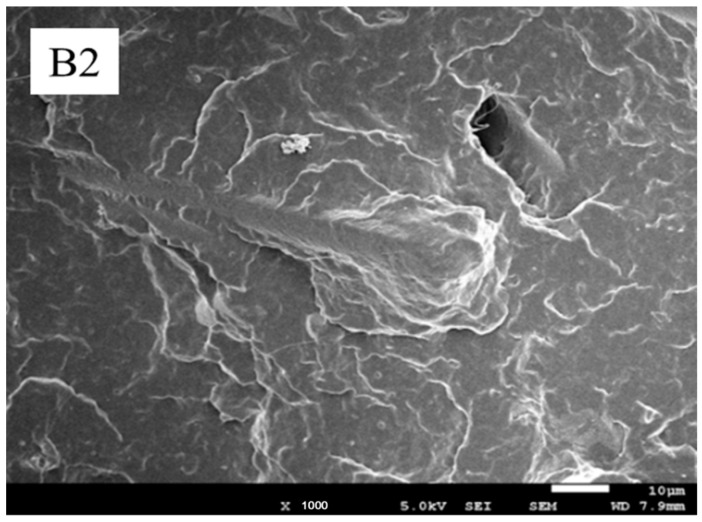
SEM images for fracture surface of impact specimen. (**A**) Before modification and (**B**) After modification.

The SEM images of the PP/N-WPCB composites before and after coating modification are shown in Figure 10. Without modification, the N-WPCB particles were unevenly distributed in the PP matrix. There were many clean glass fibers, bright holes, and smooth channels on the fracture surface of the impact specimen. All of these indicated that the N-WPCB particles without modification did not greatly enhance the PP matrix. After the N-WPCB particles were modified by KH-550, the N-WPCB particles were evenly distributed in the PP matrix. There was a lot of PP matrix stuck on the fiberglass accompanied by an obvious laddering phenomenon. This indicated that interfacial bonding between the PP matrix and N-WPCB particles could be improved effectively by KH-550 modification. The mechanical properties of PP/N-WPCB composites were also enhanced. The SEM picture of N-WCPB particle is given in Figure 1. The main components of the non-metallic printed circuit board were glass fiber and epoxy resin. As shown in Figure 1, the thermosetting resins and glass fiber separated after crushing. Due to the thermal stability of thermosetting plastics, the glass fiber should be wrapped by the PP matrix in the SEM picture of the fracture surface of the impact specimen for the PP/N-WPCB composites after coating modification.

#### 3.6.2. Infrared Analysis of the N-WPCBs Particles

**Figure 11:**
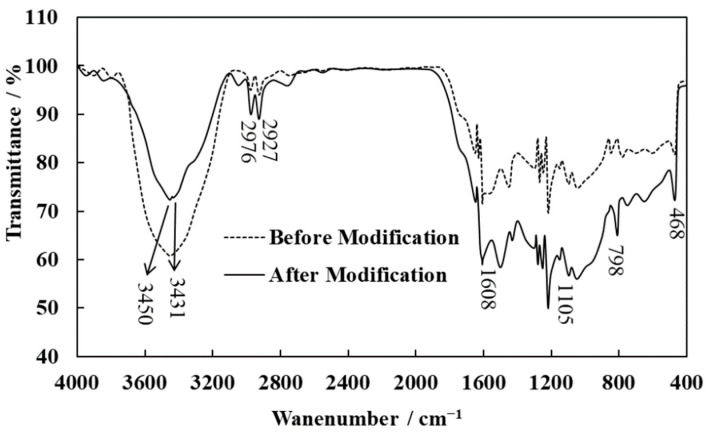
FT-IR spectra of N-WPCBs and N-WPCBs modified by KH-550.

The FT-IR spectra of N-WPCB particles before and after modification are shown in Figure 11. The strong peak at 3456 cm^−1^ is associated with –OH stretching vibrations in either Si–OH groups or physically adsorbed water on the surface of the particles. The absorptions at 1105 cm^−1^, 798 cm^−1^, and 468 cm^−1^ are caused by asymmetric stretching vibrations, symmetric stretching vibrations, and flexural vibrations of Si–O–Si, respectively. The absorptions at 2964 cm^−1^ and 2927 cm^−1^ are caused by asymmetric stretching vibrations and symmetric stretching vibrations of –CH_2_, respectively. Specifically, for the N-WPCB modified particle, the modification of KH-550 is indicated by the new absorption peaks at 3398 cm^−1^ and 1608 cm^−1^, which are caused by the stretching vibrations and flexural vibrations of –NH_2_, respectively.

According to the FT-IR results, the absorption peak of –OH vibrations became weaker after modification, while the absorption peak of Si–O–Si vibrations grew stronger. On the one hand, Si–O–Si groups were formed by Si–OH groups on the surface of N-WPCBs, which chemically reacted with Si–OH groups produced by KH-550 hydrolysis [33]. On the other hand, Si–O–Si groups formed from the associated reaction of the Si–OH groups produced by KH-550 hydrolysis [34]. New reticular membranes or organic long chains on the surface of the modified particles were formed from the associated reaction of hydrolyzed KH-550. A reticular membrane led to an organic surface of the N-WPCBs and decreased the polarity of the N-WPCBs, which enhanced the compatibility of the PP matrix and N-WPCB particles [10]. Interfacial bonding between the PP matrix and N-WPCB particles was enhanced through the organic long chains. All of this led to the development of the mechanical properties of PP/N-WPCB composites.

### 3.7. Coating Modification with Different Methods

Experimental comparative study and analysis were used for the different modification methods. The N-WPCB particles were modified using a high-speed mixer and spout-fluid bed. The composites were prepared with the modified N-WPCBs through different modification methods. The effects of modification methods on the mechanical properties of PP/N-WPCB composites are shown in Table 1. The flexural strength of the composite improved as unmodified N-WPCBs were added, while the tensile strength and notched impact decreased. The mechanical properties of the composites all improved with the three different coating modification methods. The method of coating in a spout-fluid bed with the assistance of PP particles showed the best modification effect. 

DSC tests were carried out to investigate the effects of addition N-WPCB on the crystallization behavior of PP. As shown in Table 1, the addition of the N-WPCB changed little in the *T*m of the PP/N-WPCB composite. However, the WPCB improved the *X*c, showing the nucleation effect of WPCB on PP. Meanwhile, the nucleating efficiency decreased with the modification. The result suggests that N-WPCB acted as nucleation agents in the PP matrix during nonisothermal crystallization of PP/N-WPCB composites [18]. The heterogeneous nucleation effect of N-WPCB can improve the crystallization capability of PP, thus improving the mechanical properties of composite. For the modified N-WPCB, the polar groups are introduced into the main chain of PP. Thus, it can destroy the regularity of PP chain structure [35], resulting in the decrease of crystallinity compared with unmodified N-WPCB.

After all, the mechanical properties of composites could be improved by modification with KH-550 in the spout-fluid bed. The assisted fluidization of PP particles further improved the modifying effects of the N-WPCBs. With a 4 phr KH-550 dose and a 100:75 mass ratio of PP to N-WPCBs, the composites showed a flexural strength of 55.46 MPa, tensile strength of 41.56 MPa, and notched impact strength of 6.20 kJ·m^−2^. The flexural strength, tensile strength, and impact strength of the composites increased by 16.60%, 23.22%, and 23.64%, respectively.

## 4. Conclusions

In this paper, N-WPCB particles were modified by coating KH-550 in a spout-fluid bed. Then, the modified N-WPCB particles were used as fillers to fabricate PP/N-WPCB composites by extrusion and injection molding. The effect of fluidization coating on the mechanical properties of PP/N-WPCB composites was studied. The cross-section morphologies and the surface functional groups were characterized by scanning electron microscopy and infrared spectroscopy, which revealed the modification mechanism.

(1) The PP particle, as an assistant, was able to improve the fluidization quality and coating effect of N-WPCBs. The method of coating in a spout-fluid bed with the assistance of PP particles showed a better modification effect compared than other coating methods. The flexural strength, tensile strength, and impact strength of the PP/N-WPCB composites first increased and then decreased with an increasing mass ratio of PP to N-WPCBs in the mixed fluidized bed.

(2) With increasing spouting gas velocity, KH-550 dose, atomization gas velocity, and bed temperature, the mechanical strength of the composites first increased and then decreased. The mechanical properties of the composites decreased with an increasing spray rate, while the modified N-WPCB particle size increased. The modified N-WPCB particle size first increased and then decreased when increasing the spouting gas velocity. With the increasing bed temperature and atomization gas velocity, the modified N-WPCB particle size decreased.

(3) The mechanical properties of the composites could be improved by the modification of KH-550 in the spout-fluid bed. The FT-IR and SEM results demonstrated that interfacial bonding between N-WPCBs and PP is enhanced by modified N-WPCBs, which can improve the mechanical properties of the composites. When the mass ratio of PP to N-WPCBs is 100:75 and the dose of KH-550 is 4 phr, the flexural strength, tensile strength, and impact strength of the composites increase by 16.60%, 23.22%, and 23.64%, respectively. This can realize the high-value utilization of N-WPCBs with coating modification in a spout-fluid bed.

## Figures and Tables

**Table 1:** Mechanical properties of PP/N-WPCB composites prepared with different methods.

Sample	Flexural Strength/ MPa	Flexural Modulus/ Gpa	Tensile Strength/ Mpa	Tensile Modulus/ Mpa	Impact Strength/ kJ·m^−2^	Melting Point *T*m/°C	Crystalline Ratio *X*c
Pure PP	32.47	1.54	37.39	0.86	6.32	164.58	0.35
without modification	48.79	2.26	33.64	1.27	5.07	163.42	0.42
High-speed mixer	50.42	2.02	37.38	1.11	5.82	163.21	0.38
spout-fluid bed	55.24	2.18	39.98	1.17	5.96	163.68	0.39
spout-fluid bed with PP assisted fluidization	55.46	2.09	41.56	1.15	6.20	164.01	0.37

## Data Availability

Not applicable.
